# Differences in Systemic IgA Reactivity and Circulating Th Subsets in Healthy Volunteers With Specific Microbiota Enterotypes

**DOI:** 10.3389/fimmu.2019.00341

**Published:** 2019-03-07

**Authors:** Christina Grosserichter-Wagener, Djawad Radjabzadeh, Hessel van der Weide, Kyra N. Smit, Robert Kraaij, John P. Hays, Menno C. van Zelm

**Affiliations:** ^1^Department Immunology, Erasmus MC, University Medical Center, Rotterdam, Netherlands; ^2^Department Internal Medicine, Erasmus MC, University Medical Center, Rotterdam, Netherlands; ^3^Department Medical Microbiology and Infectious Diseases, Erasmus MC, University Medical Center, Rotterdam, Netherlands; ^4^Department Immunology and Pathology, Central Clinical School, Monash University and The Alfred Hospital, Melbourne, VIC, Australia

**Keywords:** intestinal microbiota, enterotypes, IgA+ B cells, γδT cells, IgA reactivity, helper-T cells, 16S sequencing

## Abstract

Changes in the intestinal microbiota have been associated with the development of immune-mediated diseases in humans. Additionally, the introduction of defined bacterial species into the mouse intestinal microbiota has been shown to impact on the adaptive immune response. However, how much impact the intestinal microbiota composition actually has on regulating adaptive immunity remains poorly understood. Therefore, we studied aspects of the adaptive immunity in healthy adults possessing distinct intestinal microbiota profiles. The intestinal microbiota composition was determined via Illumina sequencing of bacterial 16S rRNA genes extracted from the feces of 35 individuals. Blood B-cell and T-cell subsets from the same individuals were studied using flow cytometry. Finally, the binding of fecal and plasma Immunoglobulin A (IgA) to intestinal bacteria (associated with health and disease) *Bacteroides fragilis, Prevotella copri, Bifidobacterium longum, Clostridium difficile*, and *Escherichia coli* was analyzed using ELISA. Unsupervised clustering of microbiota composition revealed the presence of three clusters within the cohort. Cluster 1 and 2 were similar to previously-described enterotypes with a predominance of *Bacteroides* in Cluster 1 and *Prevotella* in Cluster 2. The bacterial diversity (Shannon index) and bacterial richness of Cluster 3 was significantly higher than observed in Clusters 1 and 2, with the *Ruminococacceae* tending to predominate. Within circulating B- and T-cell subsets, only Th subsets were significantly different between groups of distinct intestinal microbiota. Individuals of Cluster 3 have significantly fewer Th17 and Th22 circulating cells, while Th17.1 cell numbers were increased in individuals of Cluster 1. IgA reactivity to intestinal bacteria was higher in plasma than feces, and individuals of Cluster 1 had significant higher plasma IgA reactivity against *B. longum* than individuals of Cluster 2. In conclusion, we identified three distinct fecal microbiota clusters, of which two clusters resembled previously-described “enterotypes”. Global T-cell and B-cell immunity seemed unaffected, however, circulating Th subsets and plasma IgA reactivity were significantly different between Clusters. Hence, the impact of intestinal bacteria composition on human B cells, T cells and IgA reactivity appears limited in genetically-diverse and environmentally-exposed humans, but can skew antibody reactivity and Th cell subsets.

## Introduction

All mucosal surfaces of the human body are colonized by microorganisms — termed the human “microbiota” (1). The number and composition of the microbiota (microbiota profile) varies greatly dependent on the microbial location on/in the human host, with variation also being observed at identical locations within different hosts (2). The total number of bacteria on/in the human host is estimated at the same order of magnitude as the total number of eukaryotic cells in the human body (~3.10^13^) (3), with the largest microbial load being found in the intestinal tract, where more than 1,000 bacterial species may be found (4). The vast majority of intestinal bacteria appear to be commensals, providing the host with otherwise inaccessible nutrients, whilst in addition, preventing colonization and translocation of pathogens within the host (1). The challenge therefore, is for the host to maintain immunological tolerance to its resident microbiota without activating inflammatory processes (5).

In the gut, immunological tolerance is thought to be maintained via for example, the prevention of bacterial translocation through the protective intestinal epithelial layer via the production of mucus, antibacterial peptides and secretory immunoglobulin A (IgA) (6). IgA is an immunoglobulin that is “specialized in mucosal protection” (7) and the most abundant antibody produced and secreted in the human body, mainly in the intestine (8). The human *IGH* locus contains two IgA subclasses, with IgA2 being more resistant to the action of secreted (neutralizing) bacterial proteases, as it has a shorter “hinge region” than IgA1 (9). IgA secreting plasma cells can be generated upon terminal differentiation of activated B cells, within organized intestinal lymphoid structures with cognate T-cell help (T-dependent; TD). Alternatively, B cells can mature into IgA secreting plasma cells following T-cell independent (TI) activation in the lamina propria (10, 11). Both pathways also generate IgA memory B cells with TD-derived B cells expressing CD27 and the TI derived B-cells being CD27-IgA+ (12). Once secreted, transcytosis of IgA across the gut epithelium transports it to the lumen of the intestine, where it is able to bind to bacteria (6). The majority of antibodies produced in the intestine are antigen-specific (13), with those derived from TI responses showing a high degree of polyreactivity (14). As is evident from studies in mice raised in sterile conditions (germ free; GF), the intestinal microbiota can shape both immunological tolerance and systemic immunity, resulting in lower IgA levels, fewer CD4+ and CD8+ T cells and fewer organized lymphoid structures (Peyer's patches) in the intestines, as well as fewer germinal centers in the spleens of GF mice (15).

High-throughput sequencing of the 16S rRNA gene of bacteria has greatly facilitated research into the inter-individual and inter-location diversity of the human microbiota (16). Inter-individual differences in the microbiota appear to be influenced by host genotype and environmental factors such as diet or antibiotic use (17–20), with alterations in the intestinal microbiota having been linked to various immunological diseases such as atopic disorders, inflammatory bowel disease (IBD), arthritis, type 1 diabetes and multiple sclerosis (MS) (21–25). Nevertheless, the existence of a “core” human microbiota has been reported, with individuals being clustered based on the composition/profile of their microbiota. With respect to the intestinal microbiota, three clusters (“Enterotypes”), have been observed, dependent on the relative predominance of the bacterial genera *Bacteroides, Prevotella*, and *Ruminococcus* (26).

Although the great majority of published microbiota studies have only described various correlations between specific microbiota profiles and disease, experimental studies using mouse models have demonstrated that changes in the intestinal microbiota can actually affect adaptive immune responses. Specifically, the introduction of specific pathogen free (SPF) bacteria into germfree (GF) mice has been shown to result in lower concentrations of interleukin 4 (IL-4), IL-5 and eosinophil numbers in an OVA-induced allergy model (27). In addition, administration of probiotic bacteria in these mice reduced serum IgE levels and Th2 related cytokines, whilst increasing FoxP3 expression in CD4+ T cells in the mesenteric lymph node (28). Other researchers showed that colonization with segmented filamentous bacteria (SFB) induced the differentiation of T helper 17 (Th17) cells and regulatory T cells (Treg) (29, 30).

However, these observations were made in inbred animal models under highly controlled conditions. Therefore, it remains unclear how well these findings translate to actual microbiota-immune interactions in humans (31, 32). To gain more insight into the relationship between the composition of the intestinal microbiota and associated human immune responses, we studied the local and systemic adaptive immune response of healthy adult volunteers harboring distinct intestinal microbiota profiles.

## Methods

### Human Subjects

Blood and feces samples of 35 adult individuals were collected after written informed consent was obtained (Table 1). All individuals completed a questionnaire to gather information about Body Mass Index (BMI), diet, habit, intake of antibiotics, and lifestyle. Individuals with gastrointestinal, auto-immune and intake of antibiotics in the last 6 months were excluded from the study. Fecal samples were either frozen immediately at −80°C or stored in the fridge overnight before transporting it to the laboratory. This study was performed in accordance with the Declaration of Helsinki and the guidelines of the Medical Ethics Committees of the Erasmus MC.

**Table 1:** Demographics of participants per cluster.

**Cluster (n)**	**Cluster 1**	**Cluster 2**	**Cluster 3**
	**(*n* = 7)**	**(*n* = 8)**	**(*n* = 20)**
Age in yrs (median; range)	21 (18–35)^**^	26 (20–46)^*^	30 (20–38)
Male Gender (*n*; %)	6 (86.2%)	6 (75.2%)	8 (40.0%)
Allergic (*n*; %)	5 (71.4%)	3 (37.6%)	10 (50.0%)
Pets in household (*n*; %)	3 (42.9%)	2 (25.0%)	7 (35.7%)
Smoking (*n*; %)	2 (28.6%)	0	1 (5.0%)
Vegetarian (*n*; %)	1 (14.3%)	0	1 (5.0%)
BMI (median; IQR)	21.04 (19.9–23.5)	22.68 (19.4–23.1)	23.03 (22–24.8)
WBC (median; IQR)	7.1 (4.9–7.5)	7.2 (5.6–10.4)	7.3 (5.7–8.6)
Specific IgE (median; IQR)	4.23 (0.1–23.6)	0.61 (0.17-22.9)	4.60 (0.02–13.5)
**CONSUME REGULARLY**			
Coffee (*n*; %)	4 (57.1%)	7 (87.7%)	13 (65.4%)
Probiotics (*n*; %)	0	0	1 (5.0%)
Alcohol (*n*; %)	5 (71.4%)	4 (50.0%)	15 (75.2%)

BMI, body mass index in kg/m^2^; WBC, white blood cell count in million cells/ml; Specific IgE reactivity against most common airborne allergens, >0.35 is considered positive; IQR, Interquartile range; yrs, years; consume regularly is defined as at least once a week.

** Cluster 3 vs. Cluster 1 and

* Cluster 3 vs. Cluster 2; significant difference calculated with Mann-Whitney U-test. ^*^P < 0.05; ^**^P < 0.01.

### Intestinal Microbiota Sequencing and Analysis

Bacterial genomic DNA was isolated from approximately 100 mg feces using the NorDiag-Arrow DNA extraction kit (Autogen, MA, United States). Next, the V3-V4 region of the 16S rRNA gene was amplified by PCR (Qiagen, Hilden, Germany) using the following forward: 5′-ACTCCTACGGGAGGCAGCAG-3′ and reverse: 5′-ACTACHVGGGTWTCTAAT-3′ primers. Subsequently, the amplicon (460 bp) was normalized using the SequalPrep Normalization kit (Thermo Fisher Scientific, Waltham, MA) and sequenced using the Illumina MiSeq platform (2X 300PE, v3) (Illumina, San Diego, CA).

16S profiles were generated using an in-house developed pipeline. Briefly, singleton reads were discarded. Subsequently, to normalize for differences in number of reads per sample, subsampling to 19,000 reads per sample was performed. Similar 16S rRNA gene amplicon sequences with a minimum cluster identity of 97% were clustered in operational taxonomic units (OTUs) using UPARSE (USEARCH, v8) (33). OTUs were then aligned to the SILVA 16S rRNA reference gene database using RDP classifier v2.2 with a confidence threshold of 0.90 (34, 35). In a quality control step OTUs with an abundance of <0.005% were deleted. This Targeted Locus Study project (BioProject PRJNA515953) has been deposited at DDBJ/EMBL/GenBank under the accession KCRL00000000. The version described in this paper is the first version, KCRL01000000. Data analysis was performed using R studio v3.4.1, Vegan v2.4-5 and Phyloseq v1.22.3 package. Samples were clustered by unsupervised hierarchical clustering using Bray-Curtis distance calculations. The robustness of Clusters were assessed by silhouette validation (36).

### Blood Sampling and Flow Cytometric Immunophenotyping

Peripheral blood (9 ml) was obtained from all 35 donors. Samples were centrifuged for 10 min at 820×g to obtain plasma, which was stored at −80°C for later use.

Absolute counts of blood CD3^+^ T cells, CD16^+^/56^+^ NK cells, and CD19^+^ B-cells were obtained with a diagnostic lyse-no-wash protocol from 50 μl blood (BD Biosciences, San Jose, CA). Following red blood cell lysis of 1–2 ml blood, detailed immunophenotyping of B and T cells was performed by 11-color flow cytometry using fluorochrome-conjugated antibodies listed in Table S1.

The following subsets were defined within total B cells (CD19+): naive B cells (CD27-CD38^dim^IgD+), Ig class switched memory B cells (CD27-CD38^dim^IgD-IgM- and CD27+CD38^dim^IgD-IgM-) and plasma blasts (CD27+CD38^hi^). Furthermore, total IgA+ class switched memory B cells, as well as IgA1+ and IgA2+ memory B cells were determined (Figure S3). Within total T cells (CD3+), CD4+, CD8+, and γδT cell lineages were defined. Within the CD4+ T-cell lineage, Treg cells (CD25+CD127–), follicular T helper (Tfh) cells (CD45RA-CXCR5+), Th1 cells (CD45RA–CCR6−CXCR3+CCR4–), Th2 (CD45RA–CCR6–CXCR3–CCR4+), Th17.1 (CD45RA–CCR6+CXCR3+CCR4–), Th17 (CD45RA–CCR6+CXCR3–CCR4+CCR10–) and Th22 cells (CD45RA–CCR6+CXCR3–CCR4+CCR10+) were identified (Figure S3). Data were acquired on an LSRFortessa (BD Biosciences) with standardized instrument settings (37) and analyzed using FacsDIVA software v8 (BD Biosciences).

### IgA Isolation From Feces and Plasma Samples

Approximately five grams of feces were collected from all study subjects and frozen at −80°C before use. Feces samples were thawed and homogenized in 25 ml extraction buffer consisting of Phosphate Buffered Saline (PBS; pH 7.4, 0.5% Tween-20, and 0.5% NaAc) and the mixtures were centrifuged for 20 min at 4°C and 1500×g. Two milliliters of supernatant was taken and 20 μl protease inhibitor cocktail (Sigma-Aldrich, Saint Louis, MO) added, with the mixture being centrifuged for 10 min at 10,000×g. The resulting homogenized feces mixtures were stored at −20°C.

IgA isolation from homogenized feces and plasma samples was performed using Mobicol Affinity Chromatography spin columns (35 μm pores; Mobitec, Göttingen, Germany) with high affinity for both the IgA1 and IgA2 subclasses. Following filling of the columns with 100 μl CaptureSelect IgA affinity resin (Thermo Fisher Scientific), either 500 μl homogenized feces or 200 μl plasma was added to the column and incubated for 1 h. After three wash steps using PBS pH 7.4, IgA was eluted with 0.1M glycine pH 3.

### Quantification of IgA Concentrations and IgA Reactivity to Bacterial Antigens

IgA concentrations in plasma and feces samples were determined by ELISA using coating flat-bottom 96-well plates overnight with 50 μl of 2 μg/ml Affinipure goat anti-human IgA α-chain (Jackson ImmunoResearch, West Grove, PA) in carbonate buffer. Subsequently, all wells were washed to remove unbound antibody. Fifty microliters of sample or IgA standard (Sigma-Aldrich) was added to each well and incubated for 2 h at room temperature. Bound antibodies were detected using peroxidase-conjugated goat anti-human IgA (Jackson ImmunoResearch) at a concentration of 0.8 μg/ml in PBS pH 7.4 with 1 mM Tris-EDTA and 0.05% Tween-20 and TMB ELISA substrate (ThermoFisher Scientific) according to manufacturer's instructions. Optical density (OD) was measured at 450 nm. For each plate, a PBS only negative control were used to obtain the background signal, which was subtracted from each sample measurement prior to calculation of the sample concentration using the standard curve in Soft-max Pro software v6.4 (Molecular Devices, Silicon Valley, CA).

To test IgA reactivity, the following bacterial strains were obtained from Leibniz Institute DSMZ-German Collection of Microorganisms and Cell Cultures: (1) *Bifidobacterium longum* (20088) - Gram positive - one of the earliest colonizers of the human intestine; (2) *Prevotella copri* (18205) - Gram negative - *Prevotella* enterotype; (3) *Clostridium difficile* (1296) - Gram positive - spore-forming potential human pathogen, associated with diarrhea, and colitis after antibiotic therapy; (4) *Bacteroides fragilis* (2151) - Gram positive - *Bacteroides* enterotype; and (5) *Escherichia coli*- Gram negative - the best understood of all microbiota microorganisms. Axenic culture of bacteria was performed under recommended conditions on CDC blood agar with Kanamycin and Vancomycin (BD Biosciences) for *P. copri* and on blood agar for all other bacteria. 100 μl bacterial suspension with OD600 of 0.5 was used to coat flat-bottom 96-well plates at 4°C overnight. 25 ng/ml sample IgA, calculated as mentioned above was used. As positive control IgA isolated from human colostrum (Sigma-Aldrich) showed reactivity to the five bacteria tested and was measured at concentrations of 25, 50, and 100 ng/ml on each plate. Absorbance was measured at 450 nm and background signal was subtracted. The absorbance of each sample was expressed relative to that of the positive control (mean OD sample/ mean OD pos ct) for normalization and to enable comparison of results from various samples and those obtained from different ELISA plates.

### Statistics

Statistical analyses were performed using the Mann-Whitney U test, χ^2^ test or Kruskal-Wallis, as indicated in the Figure legends. *P* < 0.05 were considered statistically significant.

## Results

### Study Cohort

Thirty-five adult volunteers were included in this study with a median age of 28 years (range 18–46 years). The study cohort consisted of 20 males and 15 females with an average BMI of 22.9. Two participants were vegetarian. Total T-, B-, and NK-cell numbers of all individuals were within the normal range. Detailed information about the study subjects can be found in Table S2.

### Three Groups of Individuals With Distinct Intestinal Microbiota Profiles

To study intestinal microbiota profiles, we collected feces and sequenced the 16S rRNA gene of extracted DNA. We obtained an average of 44,708 reads (SD = 11,905; range = 19,699−78,703 reads) per sample and identified in total 1,324 OTUs. Subsampling of 19,000 reads per sample yielded a total of 677 OTUs. The predominant phyla in our cohort feces samples were *Bacteroidetes* and *Firmicutes*, in line with previous studies (2, 38). Within *Bacteroidetes, Bacteroidaceae*, and *Prevotellaceae* were the dominant bacterial families, with the most abundant bacterial families within the *Firmicutes* being the *Lachnospiraceae* and *Ruminococcaceae*. Further classification to the genus level revealed 228 distinct genera of which 57% could be assigned to a genus. The remaining 43% were assigned to a higher taxonomic level such as the family, order or class.

**Figure 1:**
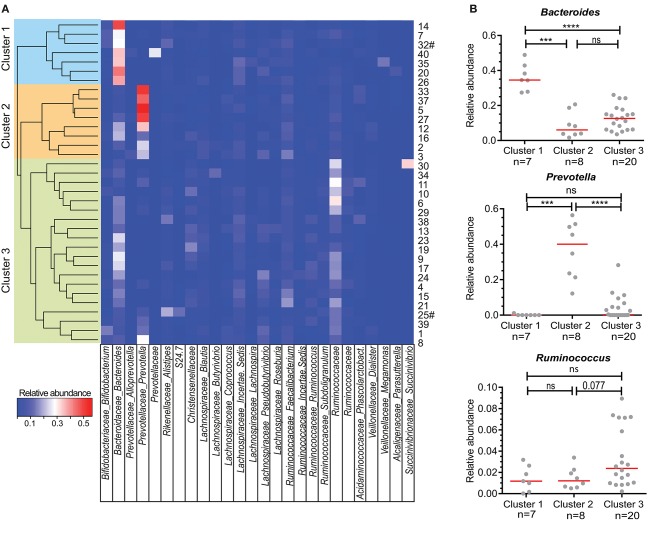
Clustering of individuals based on their microbiota composition. **(A)** Unsupervised hierarchical clustering based on Bray Curtis dissimilarity of 35 individuals using all detected bacterial genera (*n* = 228). Relative abundance of the most abundant genera are shown in the heatmap. When genus name was not assigned, the family name is given. A cluster of related microbiota profiles was determined as a cut-off value at height 0.7 of the dissimilarity index. Sample numbers given on the right # indicate vegetarian individuals. **(B)** Relative abundance of *Bacteroide*s, *Prevotella*, and *Ruminococcus* for individuals within each of the three main clusters defined in A. Each dot represents one individual, red lines are median values. Statistics were calculated with Mann-Whitney *U-*test. ^***^P < 0.001; ^****^P < 0.0001.

The abundance of these 228 genera was used to perform unsupervised hierarchical clustering with complete linkage based on Bray-Curtis dissimilarity distance (Figure 1A), and three distinct clusters were determined by calculating the average silhouette (36) (Figure S1). The median BMI, White Blood Cell count (WBC), % of males and allergic individuals did not significantly differ between the three clusters. The median age of Cluster 3 is significantly higher than that of Cluster 1 and 2 with *P* < 0.01 and *P* < 0.05, respectively (Table 1). Dietary intake, e.g., vegetarian diet, consumption of coffee, probiotics, or alcohol did not differ between the three clusters, nor did other lifestyle and environmental factors, such as smoking or pets in the household. The clustering was not grossly affected by the inclusion of the two vegetarians in the study cohort, as was shown by unsupervised clustering after exclusion of vegetarian individuals (Figure S2).

In previous studies, individuals could be clustered into three distinct enterotypes (26), which was based on the most dominant bacteria present in their feces, namely *Bacteroides, Prevotella* and *Ruminococcus*. Within our own cohort, individuals in “Cluster 1” had significantly more *Bacteroides* in their feces than individuals in Clusters 2 and 3 (Figure 1B). On the other hand, *Prevotella* was significantly more abundant in Cluster 2 than in Clusters 1 and 3. Finally, although *Ruminococcus* was not significantly higher in abundance in Cluster 3 individuals (Figure 1B), a trend could be seen with increased numbers of *Ruminoccocaceae*.

**Figure 2:**
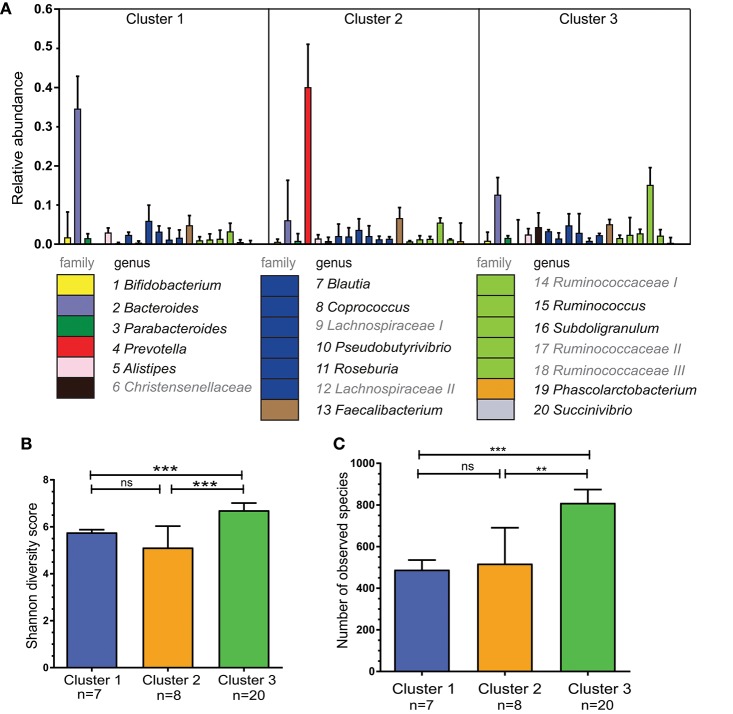
Bacterial diversity differs between the three specified clusters. **(A)** Relative abundance per cluster of the 20 most abundant bacteria present in our cohort. Each column shows the median plus interquartile range of the relative abundance of bacterial genera (or if not identified bacterial family), relative abundance of 1.0 = 100%. The numbers below indicate corresponding genus, colors show the bacterial family of the various genera. *Ruminococcaceae* I, II, and III and *Lachnospiraceae* I and II could not be assigned to different genera, but differ in OTU clusters. **(B)** Shannon diversity index for each of the three clusters to determine bacterial diversity. **(C)** Bacterial richness - defined as the total number of observed unique species per cluster. Bars represent median values with interqartile range. Statistics were calculated with Mann-Whitney U test. ^**^P < 0.01; ^***^P < 0.001.

To further characterize the composition of the microbiota within the three clusters detected, we selected the 20 most abundant genera in our cohort and quantified these per cluster (Figure 2A). The second most abundant genus in Cluster 1 was from the *Lachnospiraceae* family with *Bacteroides* also being highly abundant in Cluster 2. Of note, *Prevotella* was undetectable in 4 out of 8 individuals from Cluster 1 and in 5 out of 20 individuals of Cluster 3. The most abundant genus in the microbiota of participants in Cluster 3 could not be assigned at the genus level, but was of the family *Ruminococcaceae* (Ruminococcaceae I; Figure 2A). *Ruminococcus* is part of the *Ruminococcaceae* family.

The three clusters differed in bacterial diversity and richness (Figures 2B,C). Cluster 3 had the most diverse microbiota with 810 unique observed species. This was higher than in Cluster 1 and 2 in which there were 489 and 518 unique species, respectively (*P* < 0.0001 and *P* < 0.01).

### Blood T Cells, Memory B Cells and Microbiota Clusters

**Figure 3:**
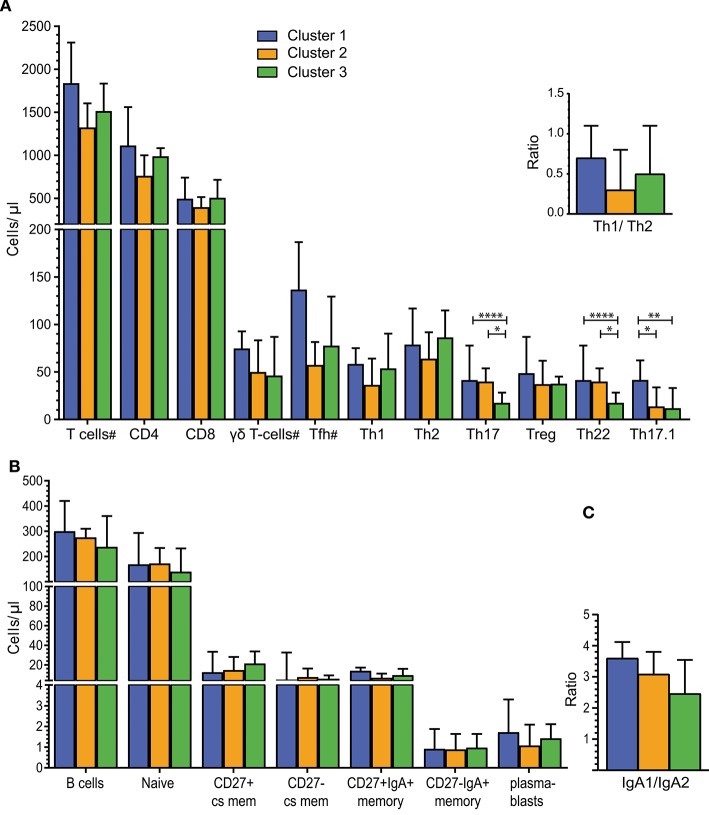
Alterations in Th cell subsets in individuals with distinct intestinal microbiota. **(A)** Absolute numbers of total T cells and T-cell subsets for Clusters 1 (*n* = 7), Cluster 2 (*n* = 7), and Cluster 3 (*n* = 20). Individuals per cluster differ for cell subsets marked with a “#” T cells: C1 = 7, C2 = 8, C3 = 20/CD4 and CD8 T cells: C1 = 7, C2 = 7, C3 = 20; γδT cells: C1 = 5, C2 = 8, C3 = 20 and Tfh: C1 = 4, C2 = 4, C3 = 11. Top right - Ratio of Th1 to Th2 cells. **(B)** B-cell counts for total B-cells and B-cell subsets for Clusters 1 (*n* = 7), Cluster 2 (*n* = 7) and Cluster 3 (*n* = 19). **(C)** Ratio of IgA1+/ IgA2+ B-cells for Cluster 1 (*n* = 5), Cluster 2 (*n* = 6), and Cluster 3 (*n* = 18). Bars in A-C represent median values with interquartile ranges. Statistics were calculated using the Mann-Whitney U test: ^*^P < 0.05; ^**^P < 0.01; ^****^P < 0.0001.

The intestinal microbiota in animal models has been shown to affect IgA+ B cells and CD8+ T cells (39) as well as CD4+ Th subset numbers (30). Therefore, T- and B-cell subsets were both analyzed in our human cohort. The total counts of CD3+ T cells, as well as CD4+, CD8+ and γδT cells, were similar between individuals of Cluster 1, 2, and 3 (Figure 3A). Multiparameter gating strategies were performed to define CD4+ T-helper cell subsets (Th1, Th2, Th17, Th17.1, Th22), and to differentiate follicular helper T cells (Tfh) and regulatory T cells (Treg) (Figure S4). Th17 and Th22 cells were significantly lower in individuals of Cluster 3 than to Clusters 1 and 2 (*P* < 0.0001 and *P* < 0.05, respectively). Th17.1 cells were significantly higher in Cluster 1 than in Clusters 2 and 3 (*P* < 0.05 and *P* < 0.01, respectively). Absolute numbers of Th1, Th2, Tfh, and Treg subsets, as well as the Th1/Th2 ratios did not differ significantly between Clusters (Figure 3A).

Considering the previously published role of secreted immunoglobulins (Igs) in intestinal immune responses, we determined the cell counts of total, naive and memory B-cells, as well as plasmablasts, in peripheral blood. Total B-cell, naive B-cell and plasmablast counts were similar in individuals with distinct microbiota composition (Figure 3B). Memory B cells were defined as CD27+ class-switched (cs; IgD-IgM-) and CD27- cs memory B cells, and within these subsets, IgA-expressing memory B cells were defined as CD27+IgA+ and CD27-IgA+ B cells. CD27-IgA+ memory B-cell numbers were lower than CD27+IgA+ memory B cells in all 3 Clusters. However, these counts did not differ significantly between the 3 Clusters (Figure 3B). To further distinguish between IgA+ memory B cells originating in the intestine or at other mucosal surfaces, we determined the expression of surface IgA1 and IgA2 on blood B cells (40). The IgA1+/IgA2+ ratios of memory B cells ranged from 2.5 to 3 and were not significantly different between the 3 Clusters (Figure 3C).

Thus, detailed blood T- and B-cell analysis showed differences in Th17, Th22, and Th17.1 cells between individuals with distinct intestinal microbiota clusters (enterotypes).

### Specific IgA to Intestinal Bacteria in Feces and Plasma

**Figure 4:**
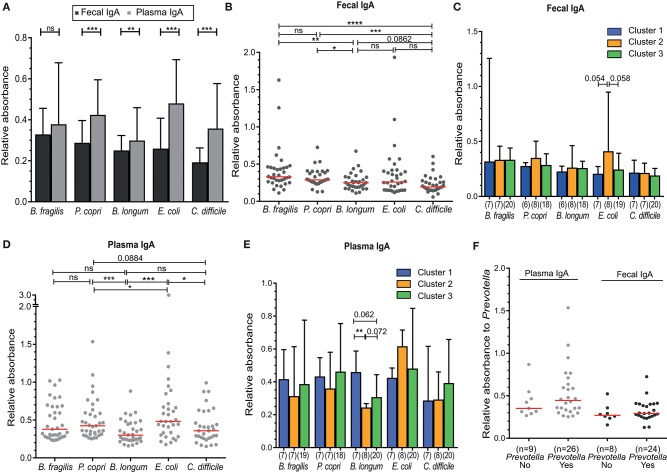
Higher IgA reactivity in plasma compared to feces against indicator microbiota organisms, but lack of a significant association to microbiota Cluster. **(A)** Fecal and plasma IgA binding against the indicator intestinal microbiota microorganisms—*Bacteroides fragilis, Prevotella copri, Bifidobacterium longum, Escherichia coli, and Clostridium difficile*. **(B)** Fecal IgA reactivity of all individuals, or **(C)** between Cluster 1–3 separately. **(D)** Plasma IgA reactivity of all individuals, or **(E)** between Clusters 1–3 separately. **(F)** Plasma and fecal IgA binding to *Prevotella copri* for individuals containing *Prevotella* (Yes) or not (No) in their intestinal microbiota. IgA binding for all experiments shown was measured as absorbance at 450 nm relative to a positive control (25 ng/ml IgA isolated from human colostrum, Sigma). Significance was determined using the Wilcoxon matched-paired signed rank test **(A)** or Mann-Whitney U test **(B,C)**. ^*^P < 0.05, ^**^P < 0.01, ^***^P < 0.001, ^****^P < 0.0001. Data represent all 35 included volunteers, which are only separated into cluster in panels **(C,E)** (cluster 1, *n* = 7; cluster 2, *n* = 8; cluster 3, *n* = 20).

For a more functional analysis of B-cell responses to the intestinal microbiota, we analyzed the binding of IgA isolated from feces and plasma samples to indicator human intestinal bacterial species, including those possessing Gram-positive and Gram-negative cell walls and associated with health and disease. These strains included the commensal bacteria *Bacteroides fragilis, Prevotella copri, Bifidobacterium longum* and the potential pathogens *Escherichia coli* and *Clostridium difficile*. Plasma IgA reactivity against all of these bacteria was higher than fecal IgA reactivity, and all differences, except for *B. fragilis*, were statistically significant (*P* < 0.05) (Figure 4A). The levels of binding of fecal IgA as assessed by relative absorbance were higher for the commensal species (*Bacteroides fragilis, Prevotella copri*, and *Bifidobacterium longum)* than for the pathogen *C. difficile* (Figure 4B). These differences were nearly all significant, with the exception of IgA binding levels between *B. longum* and *C. difficile* (*P* = 0.066). There was a trend in higher fecal IgA binding to *E. coli* in individuals of Cluster 2, but this was not significant (C1 vs. C2, *P* = 0.054; C2 vs. C3, *P* = 0.058; Figure 4C). Further, fecal IgA reactivity levels did not correlate to blood CD27+ and CD27-IgA+ memory B-cell counts (Figure S5).

Relative plasma IgA binding was highest for *E. coli*, in contrast to fecal IgA (Figure 4D). IgA binding to *B. longum* was lower than binding to the other two commensal bacteria (*B. fragilis* and *P. copri*) in both feces (*P* = 0.0016 and *P* = 0.0182) and plasma (*P* = 0.0105 and *P* = 0.0009). Plasma IgA binding to *B. longum* was lower in individuals of Cluster 2, which was significant when compared to Cluster 1 (*P* < 0.01) and not significant when compared to Cluster 3 (*P* = 0.072) (Figure 4E).

The abundance of *Prevotella* species differed greatly between individuals, with levels below our protocol detection limit in 11 cases (<0.005%) to ~55% of reads (Figures 1B, 2A). Therefore, we directly compared *Prevotella*-specific IgA between 11 individuals that showed undetectable levels of *Prevotella* to that of *Prevotella*-carriers. Both fecal and plasma IgA reactivity did not differ between individuals with (yes) or without (no) *Prevotella* as part of their intestinal microbiota (Figure 4F).

Taken together, our results demonstrate that IgA reactivity to specific intestinal bacteria is detectable in both the feces and blood plasma of healthy individuals. However, the plasma IgA levels appear to be higher than fecal IgA levels for all 5 fecal indicator microorganisms. In addition, the levels of specific IgA to *B. longum* were significantly lower in individuals of Cluster 2 as compared to individuals of Cluster 1.

## Discussion

As a first step in tackling the complexity of examining effects of intestinal microbiota profiles on adaptive immunity, we studied the immune response to 5 indicator bacteria and T- and B-cell subsets in individuals grouped in 3 defined microbiota profile clusters. Using this approach, we provide further evidence that the fecal microbiota composition from healthy individuals may be grouped into three clusters, characterized by a dominance of the *Bacteroides* or *Prevotella* genera or the *Ruminococcus* family as previously reported (26). Whereas, no differences were apparent in the B-cell compartment, Th subsets were different between the clusters with Th17 and Th22 cell numbers being lower in Cluster 3 (*Ruminococcus* enterotype), and Th17.1 cells significantly higher in Cluster 1 (*Bacteroides* enterotype). Intestinal IgA reactivity was similar between individuals with distinct intestinal microbiota, whereas plasma IgA reactivity to *B. longum* was reduced in individuals of Cluster 2 (*Prevotella* enterotype).

We found three microbiota clusters, characterized by a dominance of the *Bacteroides* or *Prevotella* genera or the *Ruminococcus* family. The classification of specific enterotypes as three clusters as initially defined by Arumugam et al. (26), is still debated. Subsequent studies since have reported two, or even four major clusters (41, 42). The commonly identified enterotypes in most publications are those clusters defined by either a higher abundance of *Bacteroides* or *Prevotella* species, with the abundance of *Prevotella* being linked to diets with a higher fiber intake and *Bacteroides* to protein-rich diets (41). Therefore, *Prevotella* and *Bacteroides* have been suggested as potentially useful biomarkers in studies determining diet and lifestyle choices in humans (43). We did not observe differences between individuals with distinct microbiome in the parameters we collected about food intake. However, we had only a limited number of questions about food intake and life style in our questionnaire and did not include questions to estimate protein or fiber intake.

In contrast to the initial description of enterotypes, a significantly higher abundance of bacteria belonging to the *Ruminococcus* genus was not observed in our cohort Cluster 3. Although the family *Ruminococcaceae* was shown to be most abundant in this Cluster, we could not be certain that this family identification comprised bacteria from the genus *Ruminococcus* or another genus from the same family. Interestingly, in previous studies the differences between *Bacteroides* and *Ruminococcus* clusters were shown to be less well-defined than originally thought, while the *Prevotella* cluster appears easier to define (44).

Despite the differences observed in microbiota profiles, we found no significant differences in blood B-cell subsets. Previously, infants colonized with *E. coli* and *Bifidobacterium* have been shown to carry more circulating CD20+CD27+ memory B cells in their blood (45). However, in our study, we did not find alterations of the B-cell compartment in blood between individuals with distinct intestinal microbiota. The composition of the intestinal microbiota in children is variable especially in the first years of life and becomes more stable in adults after 3–5 years (46). This might explain why we did not observe the types of differences previously reported in infants when using our adult cohort.

We found reduced Th17.1 cells in individuals of Cluster 2 and 3, as well as lower Th17 and Th22 cells in individuals of Cluster 3. Studies using mouse models have shown that segmented filamentous bacteria induce Th17 cell development (30, 47). In humans, Th17 cells have been described in the pathogenesis of IBD (48), and reduced Th22 cells were found in inflamed colon tissue of IBD patients (49). In contrast, Th22 and Th17 cells were increased in rheumatoid arthritis (RA)(50). Interestingly, in newly diagnosed RA patients *Prevotella* bacteria were increased and *Bacteroides* decreased. More specifically, the species *Prevotella copri* was found in 75% of RA patients, while this was only found in 21% of healthy individuals (24). In healthy controls we did not found alterations in *Prevotella* linked to Th17 and Th22 cells.

The balance between Th17 and Treg cells is influenced by the microbiome and important for the homeostasis in the gut (51). Han and co-workers have shown that Treg cell numbers were positively correlated to the abundance of *Ruminococcaceae* in the intestine of patients with graft vs. host disease. However, a decrease in Th17 cells with increased *Ruminococcaceae* abundance was not statistically significant (51). In our cohort were Th17 cells lower in individuals of Cluster 3 (*Ruminococcaceae* cluster), which confirms the trend observed by Han and co-workers. We did not find a difference in Treg cell numbers between individuals with high (Cluster 3) and low (Cluster 1 and 2) *Ruminococcaceae* abundance. Thus, in our cohort reductions in Th17- and Th22-cell numbers were not linked to increased Treg numbers, although it is possible that intestinal cell numbers may differ at specific local sites in the intestine.

Possible immunomodulatory capacities related to *Ruminococcu*s and *P. copri* have not yet been fully described, although polysaccharide A (PSA) from the capsule of *B. fragilis* have been shown to affect systemic Th1/Th2 ratios in mice (52). Additionally, Round et al. showed that PSA can alter T-cell differentiation via the induction of Tregs (53). However, profound differences exist between the microbiota and immune responses between mice and humans, which may limit the translation of mouse microbiota studies into the human situation (54). In our cohort, we found no statistically significant alterations in Treg numbers or Th1/Th2 ratios in individuals with a high abundance of *Bacteroides* in their feces. This may be explained by the fact that we studied a relatively small cohort, which might not have been sufficient to detect more subtle differences in the immune response and microbiota profiles of human populations that are exposed to many different interpersonal and environmental influences. Further, we were not able to identify *Bacteroides* at the species level, and were consequently not able to examine differences in the abundance of *B. fragilis* between individuals.

Not only bacterial colonization, but also persistent infections with herpes viruses, esp. CMV and EBV, have been shown to affect memory T-cell, but not B-cell numbers in peripheral blood (55–57). These effects included the CD4, CD8, and γδT cell lineages in CMV positive individuals. As we did not determine viral seropositivity in our study cohort, we cannot exclude a confounding effect of CMV infection. However, in our experience (56), as well as in publications from others, it is evident that >80–90% of adults are seropositive for CMV. Hence, it is unlikely that the difference in CMV serostatus would have affected the changes in T-cell numbers that we observed.

IgA reactivity to 5 indicator bacterial strains was higher in plasma than in fecal matter, although differences between individuals with distinct microbiota composition were only found for plasma IgA reactivity to *B. longum*. A potential explanation for higher plasma IgA reactivity as compared to fecal IgA could be that IgA in the intestine binds commensal bacteria with low affinity and in a polyreactive manner, possibly leading to weaker binding in our ELISA assays. In contrast, systemic immune activation may lead to strong IgA responses in peripheral blood. For example, it has been shown that recombinant IgA from intestinal IgA memory B-cells is not only polyreactive to antigens such as insulin, dsDNA and LPS, but also against different commensal bacteria (14, 58).

A correlation between IgA and the abundance of the intestinal bacteria was not found. Although the relative bacterial 16S rRNA gene quantities calculated are dependent on the number of 16S rRNA gene copies per bacterial species, we did not correct for differences in gene copy numbers between all possible fecal bacteria detected. This was due to the fact that gene copy numbers vary per individual bacterial species and current limitations in Illumina sequencing technology mean that it is not possible to accurately identify bacteria to the species level. Therefore, differences in 16S rRNA gene copy numbers could have theoretically influenced our results and masked a potential correlation between bacterial species abundance and IgA reactivity. Additionally, our ELISA methodology utilized a single representative species of 5 different bacterial genera for IgA determination, and correlations of IgA activity were obtained using only a single species from each genus under investigation.

Interestingly, we found *Prevotella*-reactive IgA antibodies in plasma and feces of individuals that apparently lacked *Prevotella* in the intestinal microbiome. There is a possibility that the abundance of this genus was below the limit of detection in our microbiome analysis. Even so, our data shows that individuals with absent or low abundance of *Prevotella* in the intestinal microbiome may still present with high levels of *P. copri*- specific IgA in feces and plasma. This is in line with studies from Hapfelmeier et al. who showed that IgA plasma cells in the intestine of mice provide long-lasting immunity, with reactivity up to of 6–8 weeks after their respective target bacteria were completely cleared from the host (59). In this respect, we only collected feces at a single time point and changes in the abundance of *Prevotella* bacteria in the feces of volunteers could have taken place over the time of the study. It would have been interesting to be able to follow our cohort in order to determine qualitative (presence/absence) changes in the abundance of *Prevotella* and other bacterial genera over time.

In our volunteers, systemic (plasma) and intestinal IgA binding to *Bifidobacterium longum* was significantly lower than to other commensals such as *Prevotella copri* and *Bacteroides fragilis*. Bifidobacteria are one of the first bacteria to colonize the intestine after birth and are beneficial for human health by their ability to skew T-helper cell responses toward Th1 response (60, 61). Further, in a study in pigs, colonization with *Bifodobacterium* and *Lactobaccilus* induced increased Rota-virus specific intestinal IgA responses after Rotavirus infection (62), though to our knowledge, we are the first to describe *Bifidobacterium*-specific IgA responses in humans.

In conclusion, we show that plasma IgA reactivity to 5 indicator microorganisms frequently found in the human intestine is higher than intestinal IgA reactivity. Systemic IgA reactivity to *B. longum* is reduced in individuals of Cluster 2 with high abundance of *Prevotella* in the intestinal microbiome. Whereas, no differences were observed in the blood B-cell compartment, Th17-, Th17.1- and Th22-cell numbers were significantly different between individuals with distinct intestinal microbiota Cluster profiles (enterotypes). Still, we have studied a small study cohort and studies involving much larger cohorts, e.g., population-based cohorts with the potential to correct for confounding factors such as lifestyle or diet will be required to confirm our results.

## Data Availability

This Targeted Locus Study project (BioProject PRJNA515953) has been deposited at DDBJ/EMBL/GenBank under the accession KCRL00000000. The version described in this paper is the first version, KCRL01000000.

## Author Contributions

CG-W and MvZ: designed research; CG-W, DR, JH, and MvZ: wrote the manuscript; CG-W, DR, HvdW, and KS: performed experiments; JH: supervised feces sample processing and storage; RK and JH: contributed to the study methodology and essential discussions of the paper. All authors critically read the manuscript and approved of the final version.

### Conflict of Interest Statement

The authors declare that the research was conducted in the absence of any commercial or financial relationships that could be construed as a potential conflict of interest.

## References

[1] Sommer F Backhed F. The gut microbiota–masters of host development and physiology. Nat Rev Microbiol. (2013) 11:227–38. 10.1038/nrmicro2974 23435359

[2] Huttenhower C Gevers D Knight R Abubucker S Badger JH Chinwalla AT Structure, function and diversity of the healthy human microbiome. Nature. (2012) 486:207–14. 10.1038/nature11234 22699609 PMC3564958

[3] Sender R Fuchs S Milo R. Revised estimates for the number of human and bacteria cells in the body. PLoS Biol. (2016) 14:e1002533. 10.1371/journal.pbio.1002533 27541692 PMC4991899

[4] Lloyd-Price J Abu-Ali G Huttenhower C. The healthy human microbiome. Genome Med. (2016) 8:51. 10.1186/s13073-016-0307-y 27122046 PMC4848870

[5] Cerf-Bensussan N Gaboriau-Routhiau V The immune system and the gut microbiota: friends or foes? Nat Rev Immunol. (2010) 10:735–44. 10.1038/nri2850 20865020

[6] Hooper LV Macpherson AJ. Immune adaptations that maintain homeostasis with the intestinal microbiota. Nat Rev Immunol. (2010) 10:159–69. 10.1038/nri2710 20182457

[7] Gutzeit C Magri G Cerutti A. Intestinal IgA production and its role in host-microbe interaction. Immunol Rev. (2014) 260:76–85. 10.1111/imr.12189 24942683 PMC4174397

[8] Kato LM Kawamoto S Maruya M Fagarasan S. Gut TFH and IgA: key players for regulation of bacterial communities and immune homeostasis. Immunol Cell Biol. (2014) 92:49–56. 10.1038/icb.2013.54 24100385

[9] Lin M Du L Brandtzaeg P Pan-Hammarström Q. IgA subclass switch recombination in human mucosal and systemic immune compartments. Mucosal Immunol. (2014) 7:511–20. 10.1038/mi.2013.68 24064668

[10] Cerutti A Rescigno M. The biology of intestinal immunoglobulin A responses. Immunity. (2008) 28:740–50. 10.1016/j.immuni.2008.05.001 18549797 PMC3057455

[11] Macpherson AJ Gatto D Sainsbury E Harriman GR Hengartner H Zinkernagel RM. A primitive T cell-independent mechanism of intestinal mucosal IgA responses to commensal bacteria. Science. (2000) 288:2222–6. 10.1126/science.288.5474.2222 10864873

[12] Berkowska MA Driessen GJ Bikos V Grosserichter-Wagener C Stamatopoulos K Cerutti A. Human memory B cells originate from three distinct germinal center-dependent and -independent maturation pathways. Blood. (2011) 118:2150–8. 10.1182/blood-2011-04-345579 21690558 PMC3342861

[13] Benckert J Schmolka N Kreschel C Zoller MJ Sturm A Wiedenmann B. The majority of intestinal IgA+ and IgG+ plasmablasts in the human gut are antigen-specific. J Clin Invest. (2011) 121:1946–55. 10.1172/JCI44447 21490392 PMC3083800

[14] Berkowska MA Schickel JN Grosserichter-Wagener C de Ridder D Ng YS van Dongen JJ. Circulating Human CD27-IgA+ Memory B cells recognize bacteria with polyreactive Igs. J Immunol. (2015) 195:1417–26. 10.4049/jimmunol.1402708 26150533 PMC4595932

[15] Round JL Mazmanian SK The gut microbiota shapes intestinal immune responses during health and disease. Nat Rev Immunol. (2009) 9:313–23. 10.1038/nri2515 19343057 PMC4095778

[16] Jovel J Patterson J Wang W Hotte N O'Keefe S Mitchel T Characterization of the gut microbiome using 16S or shotgun metagenomics. Front Microbiol. (2016) 7:459 10.3389/fmicb.2016.00459 27148170 PMC4837688

[17] Dethlefsen L Relman DA. Incomplete recovery and individualized responses of the human distal gut microbiota to repeated antibiotic perturbation. Proc Natl Acad Sci USA. (2011) 108 (Suppl. 1):4554–61. 10.1073/pnas.100008710 20847294 PMC3063582

[18] Turnbaugh PJ Hamady M Yatsunenko T Cantarel BL Duncan A Ley RE. A core gut microbiome in obese and lean twins. Nature. (2009) 457:480–4. 10.1038/nature07540 19043404 PMC2677729

[19] Ubeda C Pamer EG. Antibiotics, microbiota, and immune defense. Trends Immunol. (2012) 33:459–66. 10.1016/j.it.2012.05.003 22677185 PMC3427468

[20] Claesson MJ Jeffery IB Conde S Power SE O'Connor EM Cusack S. Gut microbiota composition correlates with diet and health in the elderly. Nature. (2012) 488:178–84. 10.1038/nature11319 22797518

[21] Penders J Stobberingh EE van den Brandt PA Thijs C. The role of the intestinal microbiota in the development of atopic disorders. Allergy. (2007) 62:1223–36. 10.1111/j.1398-9995.2007.01462.x 17711557

[22] Jangi S Gandhi R Cox LM Li N von Glehn F Yan R. Alterations of the human gut microbiome in multiple sclerosis. Nat Commun. (2016) 7:12015. 10.1038/ncomms12015 27352007 PMC4931233

[23] Kostic AD Gevers D Siljander H Vatanen T Hyötyläinen T Hämäläinen AM. The dynamics of the human infant gut microbiome in development and in progression toward type 1 diabetes. Cell Host Microbe. (2015) 17:260–73. 10.1016/j.chom.2015.01.001 25662751 PMC4689191

[24] Scher JU Sczesnak A Longman RS Segata N Ubeda C Bielski C. Expansion of intestinal Prevotella copri correlates with enhanced susceptibility to arthritis. Elife. (2013) 2:e01202. 10.7554/eLife.01202 24192039 PMC3816614

[25] Walters WA Xu Z Knight R. Meta-analyses of human gut microbes associated with obesity and IBD. FEBS Lett. (2014) 588:4223–33. 10.1016/j.febslet.2014.09.039 25307765 PMC5050012

[26] Arumugam M Raes J Pelletier E Le Paslier D Yamada T Mende DR. Enterotypes of the human gut microbiome. Nature. (2011) 473:174–80. 10.1038/nature09944 21508958 PMC3728647

[27] Herbst T Sichelstiel A Schär C Yadava K Bürki K Cahenzli J. Dysregulation of allergic airway inflammation in the absence of microbial colonization. Am J Respir Crit Care Med. (2011) 184:198–205. 10.1164/rccm.201010-1574OC 21471101

[28] Feleszko W Jaworska J Rha RD Steinhausen S Avagyan A Jaudszus A. Probiotic-induced suppression of allergic sensitization and airway inflammation is associated with an increase of T regulatory-dependent mechanisms in a murine model of asthma. Clin Exp Allergy. (2007) 37:498–505. 10.1111/j.1365-2222.2006.02629.x 17430345

[29] Gaboriau-Routhiau V Rakotobe S Lécuyer E Mulder I Lan A Bridonneau C. The key role of segmented filamentous bacteria in the coordinated maturation of gut helper T cell responses. Immunity. (2009) 31:677–89. 10.1016/j.immuni.2009.08.020 19833089

[30] Ivanov II Atarashi K Manel N Brodie EL Shima T Karaoz U. Induction of intestinal Th17 cells by segmented filamentous bacteria. Cell. (2009) 139:485–98. 10.1016/j.cell.2009.09.033 19836068 PMC2796826

[31] Masopust D Sivula CP Jameson SC. Of Mice, Dirty Mice, and Men: using mice to understand human immunology. J Immunol. (2017) 199:383–8. 10.4049/jimmunol.1700453 28696328 PMC5512602

[32] Tao L Reese TA. Making mouse models that reflect human immune responses. Trends Immunol. (2017) 38:181–93. 10.1016/j.it.2016.12.007 28161189

[33] Edgar RC. UPARSE: highly accurate OTU sequences from microbial amplicon reads. Nat Methods. (2013) 10:996–8. 10.1038/nmeth.2604 23955772

[34] Wang Q Garrity GM Tiedje JM Cole JR. Naive Bayesian classifier for rapid assignment of rRNA sequences into the new bacterial taxonomy. Appl Environ Microbiol. (2007) 73:5261–7. 10.1128/AEM.00062-07 17586664 PMC1950982

[35] Quast C Pruesse E Yilmaz P Gerken J Schweer T Yarza P. The SILVA ribosomal RNA gene database project: improved data processing and web-based tools. Nucl Acids Res. (2013) 41:D590–6. 10.1093/nar/gks1219 23193283 PMC3531112

[36] Kaufman LRP Finding Groups in Data: An Introduction To Cluster Analysis. New York, NY: John Wiley (1990).

[37] Kalina T Flores-Montero J van der Velden VH Martin-Ayuso M Böttcher S Ritgen M. Euroflow standardization of flow cytometer instrument settings and immunophenotyping protocols. Leukemia. (2012) 26:1986–2010. 10.1038/leu.2012.122 22948490 PMC3437409

[38] Qin J Li R Raes J Arumugam M Burgdorf KS Manichanh C. A human gut microbial gene catalogue established by metagenomic sequencing. Nature. (2010) 464:59–65. 10.1038/nature08821 20203603 PMC3779803

[39] Potockova H Sinkorova J Karova K Sinkora M. The distribution of lymphoid cells in the small intestine of germ-free and conventional piglets. Dev Comp Immunol. (2015) 51:99–107. 10.1016/j.dci.2015.02.014 25743381

[40] Blanco E Pérez-Andrés M Arriba-Méndez S Contreras-Sanfeliciano T Criado I Pelak O. Age-associated distribution of normal B-cell and plasma cell subsets in peripheral blood. J Allergy Clin Immunol. (2018) 141:2208–19 e16. 10.1016/j.jaci.2018.02.017 29505809

[41] Wu GD Chen J Hoffmann C Bittinger K Chen YY Keilbaugh SA. Linking long-term dietary patterns with gut microbial enterotypes. Science. (2011) 334:105–8. 10.1126/science.1208344 21885731 PMC3368382

[42] Costea PI Hildebrand F Arumugam M Bäckhed F Blaser MJ Bushman FD Enterotypes in the landscape of gut microbial community composition. Nat Microbiol. (2018) 3:8–16. 10.1038/s41564-017-0072-8 29255284 PMC5832044

[43] Gorvitovskaia A Holmes SP Huse SM. Interpreting prevotella and bacteroides as biomarkers of diet and lifestyle. Microbiome. (2016) 4:15. 10.1186/s40168-016-0160-7 27068581 PMC4828855

[44] Yong E Gut microbial 'enterotypes' become less clear-cut. Nature (2012). 10.1038/nature.2012.10276

[45] Lundell AC Björnsson V Ljung A Ceder M Johansen S Lindhagen G. Infant B cell memory differentiation and early gut bacterial colonization. J Immunol. (2012) 188:4315–22. 10.4049/jimmunol.1103223 22490441

[46] Rodríguez JM Murphy K Stanton C Ross RP Kober OI Juge N. The composition of the gut microbiota throughout life, with an emphasis on early life. Microb Ecol Health Dis. (2015) 26:26050. 10.3402/mehd.v26.26050 25651996 PMC4315782

[47] Ivanov II Frutos Rde L Manel N Yoshinaga K Rifkin DB Sartor RB. Specific microbiota direct the differentiation of IL-17-producing T-helper cells in the mucosa of the small intestine. Cell Host Microbe. (2008) 4:337–49. 10.1016/j.chom.2008.09.009 18854238 PMC2597589

[48] Maloy KJ Powrie F. Intestinal homeostasis and its breakdown in inflammatory bowel disease. Nature. (2011) 474:298–306. 10.1038/nature10208 21677746

[49] Leung JM Davenport M Wolff MJ Wiens KE Abidi WM Poles MA. IL-22-producing CD4+ cells are depleted in actively inflamed colitis tissue. Mucosal Immunol. (2014) 7:124–33. 10.1038/mi.2013.31 23695510 PMC3870042

[50] Zhang L Li YG Li YH Qi L Liu XG Yuan CZ. Increased frequencies of Th22 cells as well as Th17 cells in the peripheral blood of patients with ankylosing spondylitis and rheumatoid arthritis. PLoS ONE. (2012) 7:e31000. 10.1371/journal.pone.0031000 22485125 PMC3317658

[51] Omenetti S Pizarro TT. The Treg/Th17 Axis: a dynamic balance regulated by the gut microbiome. Front Immunol. (2015) 6:639. 10.3389/fimmu.2015.00639 26734006 PMC4681807

[52] Mazmanian SK Liu CH Tzianabos AO Kasper DL. An immunomodulatory molecule of symbiotic bacteria directs maturation of the host immune system. Cell. (2005) 122:107–18. 10.1016/j.cell.2005.05.007 16009137

[53] Round JL Mazmanian SK. Inducible Foxp3+ regulatory T-cell development by a commensal bacterium of the intestinal microbiota. Proc Natl Acad Sci USA. (2010) 107:12204–9. 10.1073/pnas.0909122107 20566854 PMC2901479

[54] Nguyen TL Vieira-Silva S Liston A Raes J. How informative is the mouse for human gut microbiota research? Dis Model Mech. (2015) 8:1–16. 10.1242/dmm.017400 25561744 PMC4283646

[55] van den Heuvel D Jansen MA Bell AI Rickinson AB Jaddoe VW van Dongen JJ. Transient reduction in IgA(+) and IgG(+) memory B cell numbers in young EBV-seropositive children: the Generation R Study. J Leukoc Biol. (2017) 101:949–56. 10.1189/jlb.5VMAB0616-283R 27821468

[56] van den Heuvel D Jansen MA Dik WA Bouallouch-Charif H Zhao D van Kester KA. Cytomegalovirus- and epstein-barr virus-induced t-cell expansions in young children do not impair naive t-cell populations or vaccination responses: the generation R Study. J Infect Dis. (2016) 213:233–42. 10.1093/infdis/jiv369 26142434

[57] Brodin P Jojic V Gao T Bhattacharya S Angel CJ Furman D. Variation in the human immune system is largely driven by non-heritable influences. Cell. (2015) 160:37–47. 10.1016/j.cell.2014.12.020 25594173 PMC4302727

[58] Bunker JJ Erickson SA Flynn TM Henry C Koval JC Meisel M. Natural polyreactive IgA antibodies coat the intestinal microbiota. Science. (2017) 358:6361. 10.1126/science.aan6619 28971969 PMC5790183

[59] Hapfelmeier S Lawson MA Slack E Kirundi JK Stoel M Heikenwalder M. Reversible microbial colonization of germ-free mice reveals the dynamics of IgA immune responses. Science. (2010) 328:1705–9. 10.1126/science.1188454 20576892 PMC3923373

[60] O'Callaghan A van Sinderen D. Bifidobacteria and Their Role as Members of the Human Gut Microbiota. Front Microbiol. (2016) 7:925. 10.3389/fmicb.2016.00925 27379055 PMC4908950

[61] Li CY Lin HC Lai CH Lu JJ Wu SF Fang SH. Immunomodulatory effects of lactobacillus and Bifidobacterium on both murine and human mitogen-activated T cells. Int Arch Allergy Immunol. (2011) 156:128–36. 10.1159/000322350 21576983

[62] Kandasamy S Chattha KS Vlasova AN Rajashekara G Saif LJ. Lactobacilli and Bifidobacteria enhance mucosal B cell responses and differentially modulate systemic antibody responses to an oral human rotavirus vaccine in a neonatal gnotobiotic pig disease model. Gut Microbes. (2014) 5:639–51. 10.4161/19490976.2014.969972 25483333 PMC4615723

