# Supplementary material for: Differences in Systemic IgA Reactivity and Circulating Th Subsets in Healthy Volunteers With Specific Microbiota Enterotypes
Source: Front Immunol. 2019 Mar 7;10:341. doi: 10.3389/fimmu.2019.00341 (PMC6417458; doi:10.3389/fimmu.2019.00341)
Supplement: Supplementary file 1 [file Data_Sheet_1.docx]

**SUPPLEMENTAL DATA (2 Tables and 2 Figures)**

**Table S1.** Antibodies used for flow cytometric immunophenotyping

| Antibody | Clone | Fluorochrome | Supplier |
| --- | --- | --- | --- |
| CD3 | UCHT1 | BV711 | BD Biosciences, San Jose, CA |
| CD3 | SK7 | BV786 | BD Biosciences |
| CD4 | OKT4 | BV510 | BioLegend, San Diego, CA |
| CD8 | SK1 | APCH7 | BD Biosciences |
| CD19 | J3-119 | PC7 | Beckman Coulter, Fullerton, CA |
| CD20 | 2H7 | BV510 | BioLegend |
| CD21 | B-ly4 | BV711 | BD Biosciences |
| CD23 | EBVCS-5 | APC | BD Biosciences |
| CD25 | BC96 | BV421 | BioLegend |
| CD25 | 2A3 | PE | BD Biosciences |
| CD27 | O323 | BV421 | BioLegend |
| CD28 | CD28.2 | PerCP-Cy5.5 | BioLegend |
| CD38 | HIT2 | BV605 | BioLegend |
| CD45RA | HI100 | BV605 | BioLegend |
| CD45RO | UCHL1 | FITC | DAKO, Glostrup, Denmark |
| CD69 | FN50 | BV711 | BD Biosciences |
| CD79B | SN8 | PerCP-Cy5.5 | BD Biosciences |
| CD80 | 2D10 | BV605 | BioLegend |
| CD86 | IT2.2 | PE-Cy7 | BioLegend |
| CD95 | DX2 | FITC | BD Biosciences |
| CD127 | A019D5 | APC | BioLegend |
| CCR4 | TG6/CCR4 | PECy7 | BioLegend |
| CCR6 | G034E3 | PerCP-Cy5.5 | BioLegend |
| CCR7 | REA108 | PE | Miltenyi Biotec, Cologne, Germany |
| CCR10 | 6588-5 | PE | R&D systems, Minneapolis, MN |
| CXCR3 | G025H7 | FITC | BioLegend |
| CXCR5 | 51505 | APC | R&D systems |
| IgA | IS11-8E10 | FITC | Miltenyi Biotec |
| IgD | IA6-2 | PECF594 | BD Biosciences |
| IgG | G18-145 | PE | BD Biosciences |
| IgM | MHM-88 | BV510 | BioLegend |
| TACI | 1A1 | APC | BD Biosciences |
| TACI | 1A1 | PE-DAZZLE | BioLegend |
| TCRγδ | 11F2 | PECy7 | BD Biosciences |
| Viability dye |  | AF700 | BD Biosciences |

**Table S2.** Characteristics of study subjects

| **subject** | **Age**  **(yrs)** | **Cluster** | **sex** | **allergy** | **vegetarian** | **BMI** | **WBC** | **specific IgE reactivity** | **B-cells/ µl** | **NK-cells/ µl** | **T-cells/ µl** | **CD4+ T cells/ µl** | **CD8+ T cells/µl** |
| --- | --- | --- | --- | --- | --- | --- | --- | --- | --- | --- | --- | --- | --- |
| 1 | 28 | 3 | Male | - | No | 23.3 | 5.8 | 4.9 | 298 | 160 | 1345 | 979 | 301 |
| 2 | 27 | 2 | Male | - | No | 23.1 | 7 | 0.67 | 276 | 409 | 1844 | 1359 | 409 |
| 3 | 29 | 2 | Female | - | No | 18.0 | 11.4 | 0.55 | 250 | 213 | 1352 | 801 | 484 |
| 4 | 30 | 3 | Female | - | No | 18.6 | 4.8 | 0.03 | 200 | 100 | 1390 | 1056 | 291 |
| 5 | 20 | 2 | Male | - | No | 22.7 | 5.1 | 0.03 | 203 | 60 | 1297 | 839 | 352 |
| 6 | 33 | 3 | Male | - | Yes | 23.0 | 7.6 | 2.88 | 420 | 190 | 2060 | 1320 | 579 |
| 7 | 20 | 1 | Male | Hay fever, HDM | Yes | 19.6 | 4.9 | 100 | 142 | 60 | 1026 | 542 | 460 |
| 8 | 35 | 3 | Female | - | No | 22.2 | 8.8 | 31.5 | 210 | 440 | 1400 | 983 | 388 |
| 9 | 28 | 3 | Female | - | No | 27.3 | 8.2 | 0.79 | 258 | 208 | 1146 | 588 | 509 |
| 10 | 36 | 3 | Female | - | No |  | 11.2 | 36.9 | 130 | 420 | 1710 | 1115 | 494 |
| 11 | 24 | 3 | Male | HDM, dog- and cat allergy | No | 21.3 | 4.7 | 1.44 | 105 | 113 | 1141 | 805 | 299 |
| 12 | 25 | 2 | Female | Hay fever | No | 19.4 | 10.4 | 0.11 | 178 | 171 | 1438 | 1022 | 385 |
| 13 | 37 | 3 | Male | - | No | 29.4 | 8.1 | 7.17 | 272 | 137 | 1838 | 1053 | 735 |
| 14 | 28 | 1 | Male | - | No | 23.5 | 4 | 0.03 | 300 | 250 | 2310 | 1769 | 485 |
| 15 | 28 | 3 | Male | - | No | 23.2 | 9.1 | 6.33 | 214 | 121 | 1291 | 687 | 501 |
| 16 | 46 | 2 | Male | Hay fever | No | ND | ND | 0.33 | 170 | 180 | 590 | 384 | 185 |
| 17 | 33 | 3 | Female | HDM, dog- and cat allergy | No | 25.9 | 8.6 | 0.02 | 137 | 151 | 1560 | 1091 | 437 |
| 19 | 27 | 3 | Female | - | No | 22.0 | 4.8 | 7.13 | 130 | 160 | 1470 | 1136 | 298 |
| 20 | 18 | 1 | Male | Hay fever, HDM | No | 21.0 | 5.8 | 8.52 | 230 | 170 | 1970 | 993 | 879 |
| 21 | 35 | 3 | Female | Hay fever, dog- and cat allergy | No | 18.9 | 3.9 | 0.24 | 250 | 220 | 1970 | 1336 | 587 |
| 23 | 28 | 3 | Male | Hay fever, dog- and cat allergy | Yes | 23.3 | 5.9 | 4.3 | 239 | 364 | 947 | 558 | 316 |
| 24 | 36 | 3 | Female | - | No | nd | ND | 0.41 | 320 | 220 | 1660 | 1142 | 466 |
| 25 | 36 | 3 | Female | Hay fever | No | 21.8 | 7.7 | 0.04 | 117 | 222 | 1801 | 1025 | 694 |
| 26 | 20 | 1 | Male | Hay fever, dog- and cat allergy | No | 23.4 | 7.2 | 4.23 | 100 | 100 | 930 | 555 | 330 |
| 27 | 24 | 2 | Male | HDM | No | 26.3 | 7.2 | 2.68 | 310 | 240 | 1660 | 865 | 704 |
| 29 | 28 | 3 | Male | Hay fever, dog allergy | No | 23.1 | 6.9 | 0.36 | 360 | 190 | 1280 | 846 | 392 |
| 30 | 38 | 3 | Female | - | No | 22.8 | 6.7 | 15.6 | 177 | 84 | 1039 | 756 | 228 |
| 32 | 23 | 1 | Female | Hay fever, HDM | No | 19.9 | 7.5 | 0.1 | 330 | 460 | 1840 | 1198 | 535 |
| 33 | 24 | 2 | Male | Hay fever, HDM, cat- and dog allergy | No | 20.2 | 5.6 | 29.6 | 330 | 390 | 1220 | 610 | 520 |
| 34 | 20 | 3 | Female | - | No | 24.4 | 7.3 | 7.19 | 390 | 390 | 1820 | 1077 | 659 |
| 35 | 21 | 1 | Male | HDM | No | 20.1 | 7.9 | 23.6 | 420 | 1070 | 2460 | 1508 | 827 |
| 37 | 27 | 2 | Male | Hay fever, HDM, cat- and dog allergy | No | 23.0 | 7.5 | 29.6 | 280 | 170 | 1070 | 687 | 325 |
| 38 | 30 | 3 | Female | - | No | 26.4 | 9.8 | 38.1 | 400 | 380 | 3050 | 1394 | 1400 |
| 39 | 28 | 3 | Male | Hay fever and cat allergy | No | 22.3 | 5.7 | 56.1 | 360 | 530 | 2100 | 1090 | 758 |
| 40 | 35 | 1 | Male | Hay fever | Yes | 24.8 | 7.1 | 0.14 | 450 | 340 | 1480 | 1091 | 355 |
| Abbreviations: BMI, body mass index; HDM, house dust mite; ND, not determined; WBC, white blood cell count | | | | | | | | | | | | | |


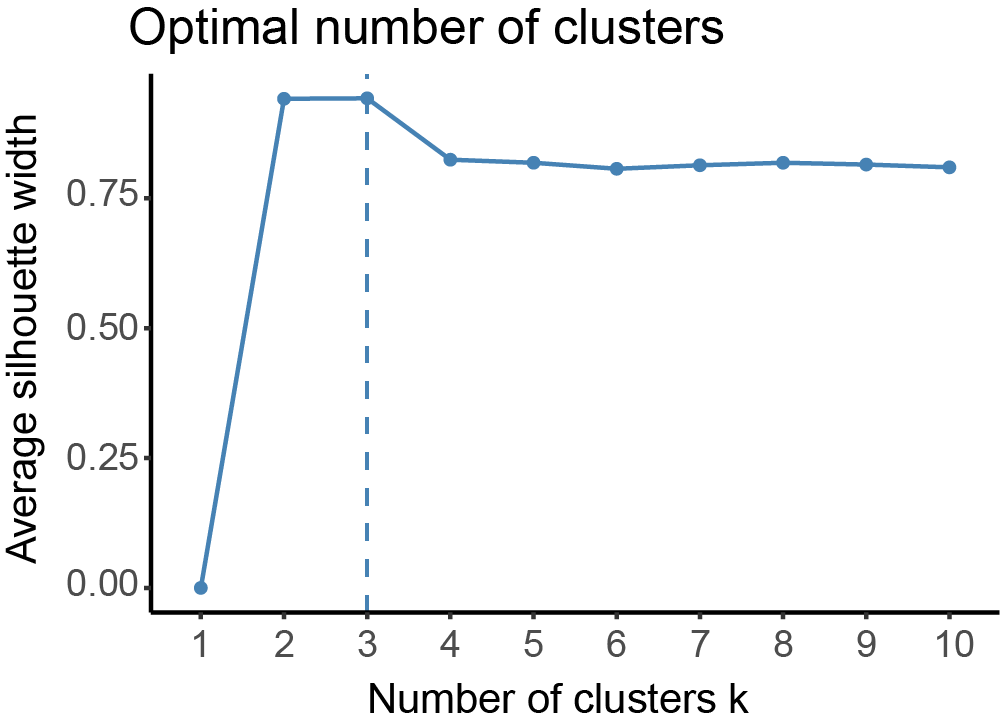


**­Figure S1: Average silhouette width of relative abundance bacterial genera.** Optimal number of clusters determined with "factoextra" package in R studio. Following function was used to obtain figure: fviz_nbclust(data1, hcut, method = "silhouette",hc_method = "complete").

_
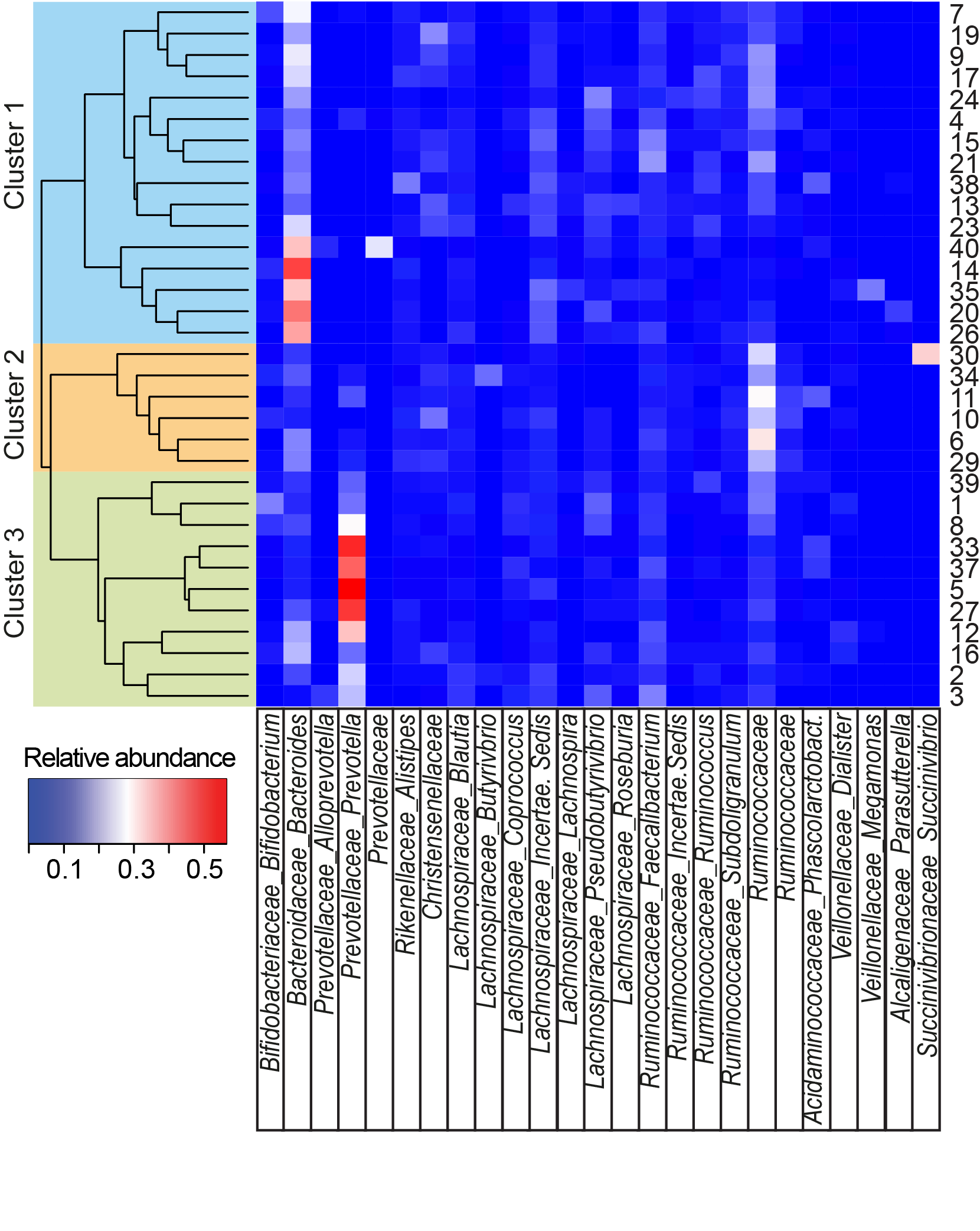
_

**Figure S2: Clustering of non-vegetarian individuals based on their microbiota composition.** Unsupervised hierarchical clustering based on Bray Curtis dissimilarity of 33 individuals using all detected bacterial genera (n=229). Relative abundance of the most abundant genera is shown in the heatmap. When genus name was not assigned, the family name is given. Sample numbers given on the right.

**
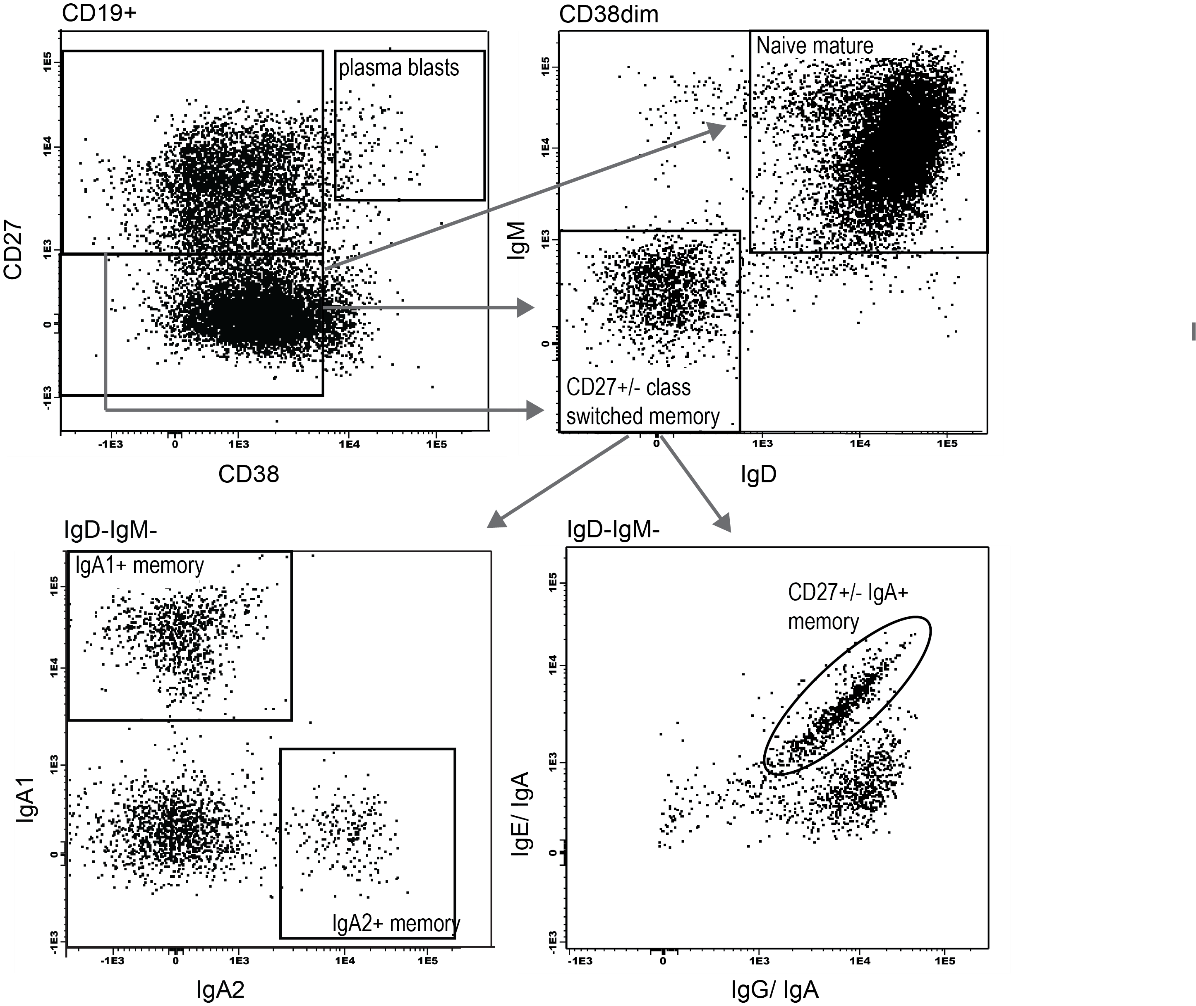
**

**Figure S3: Gating strategy of B cell subsets**. B cells were gated as CD19+ lymphocytes, and within these the following subsets were defined: plasma blasts (CD27+CD38^hi^), naive mature B cells (CD27-CD38^dim^IgD+IgM+), CD27- and CD27+ Ig class switched memory B cells (CD38^dim^IgD-IgM-), CD27- and CD27+IgA memory B cells (CD38^dim^IgD-IgM-IgA+) and IgA1+ (CD38^dim^IgD-IgM-IgA1+) and IgA2+ memory B cells (CD38^dim^IgD-IgM-IgA2+).

**
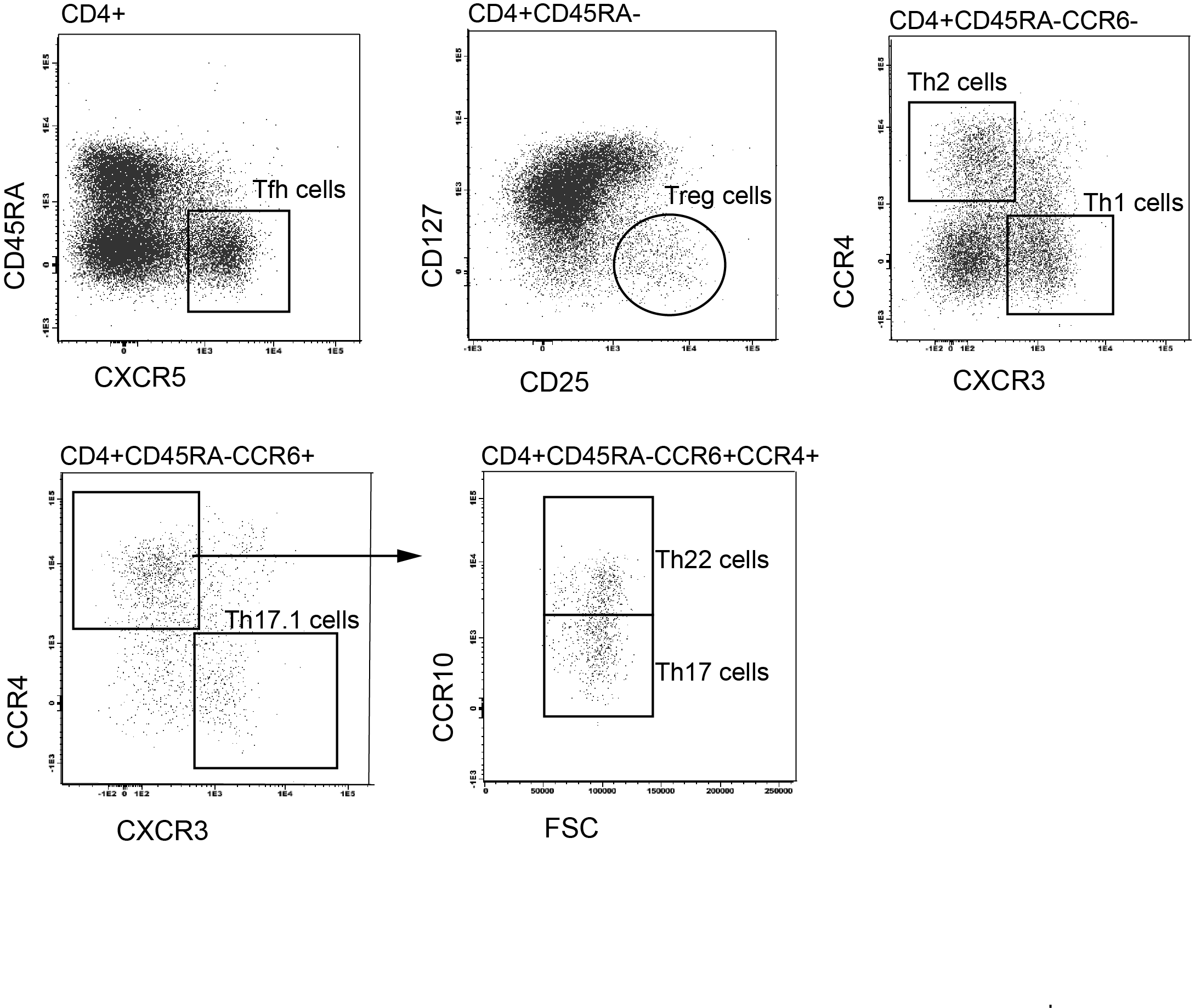
**

**Figure S4: Gating strategy of T-helper cell subsets**. CD3+CD8-CD4+ T cells were gated as indicated to determine Treg and Tfh. In addition, Th1 and Th2 gating was performed within the CCR6- cell population. Th17.1, Th17 and Th22 were defined as being CCR6+. Th17 and Th22 cells were discriminated based on differential expression of CCR10.

**
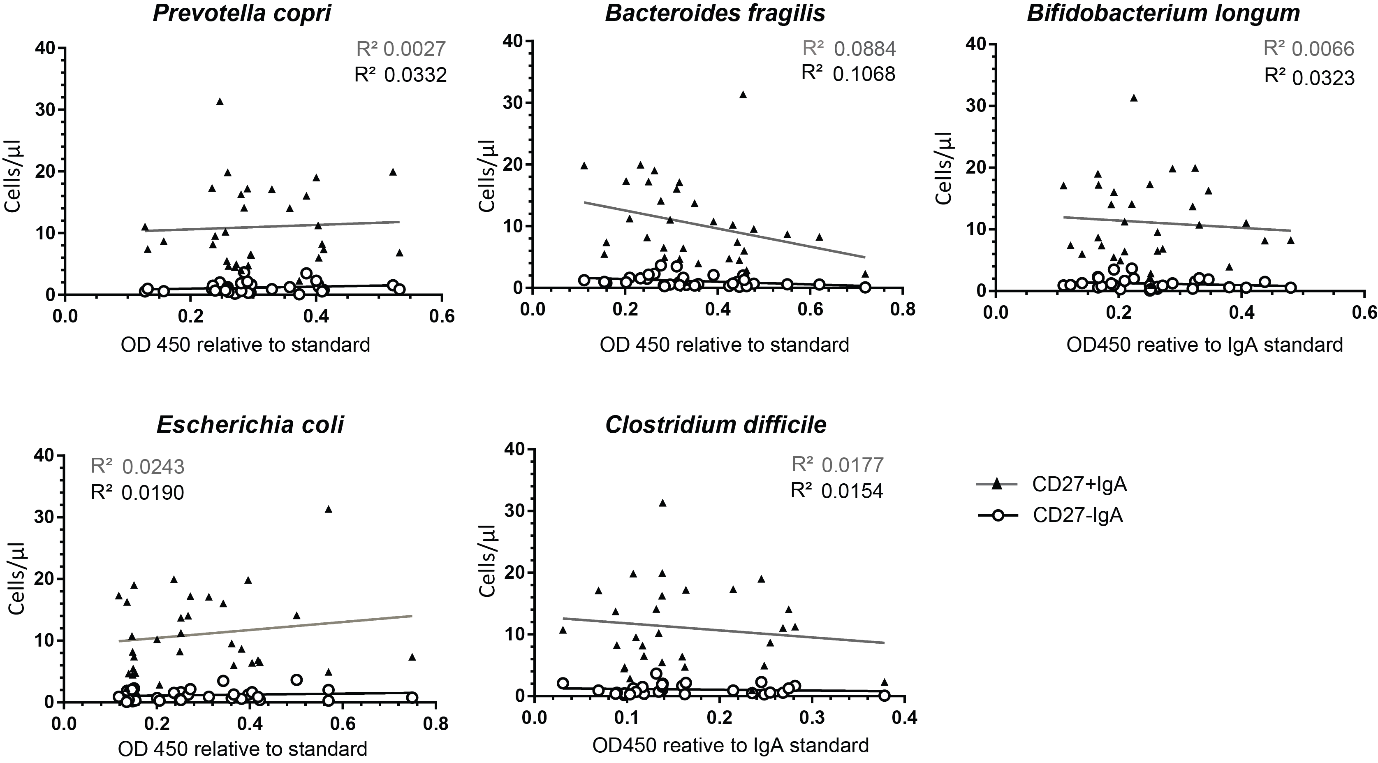
**

**Figure S5:** Correlation of fecal IgA reactivity to indicator intestinal bacteria as measured by ELISA to IgA+ memory B-cell numbers indicated as cells/µl. Δ represent CD27+IgA+ memory B-cells; O represent CD27-IgA+ memory B-cells.
